# ccbmlib – a Python package for modeling Tanimoto similarity value distributions

**DOI:** 10.12688/f1000research.22292.2

**Published:** 2020-03-05

**Authors:** Martin Vogt, Jürgen Bajorath

**Affiliations:** 1 Department of Life Science Informatics, B-IT, University of Bonn, Endenicher Allee 19c, Bonn, NRW, 53115, Germany

**Keywords:** Bernoulli model, fingerprints, p-value, similarity value distributions, Tanimoto coefficient.

## Abstract

The ccbmlib Python package is a collection of modules for modeling similarity value distributions based on Tanimoto coefficients for fingerprints available in RDKit. It can be used to assess the statistical significance of Tanimoto coefficients and evaluate how molecular similarity is reflected when different fingerprint representations are used. Significance measures derived from *p*-values allow a quantitative comparison of similarity scores obtained from different fingerprint representations that might have very different value ranges. Furthermore, the package models conditional distributions of similarity coefficients for a given reference compound. The conditional significance score estimates where a test compound would be ranked in a similarity search. The models are based on the statistical analysis of feature distributions and feature correlations of fingerprints of a reference database. The resulting models have been evaluated for 11 RDKit fingerprints, taking a collection of ChEMBL compounds as a reference data set. For most fingerprints, highly accurate models were obtained, with differences of 1% or less for Tanimoto coefficients indicating high similarity.

## Introduction

The quantitative assessment of molecular similarity is a central concept in chemoinformatics ^1– 4^. It forms the basis of similarity searching and ligand-based virtual screening to identify novel molecules in large databases with biological properties similar to given reference compounds ^5– 7^. Assessment of molecular similarity plays a central role in chemical space analysis and the study of activity landscapes where chemical space projections onto low-dimensional representations are based on quantified similarities ^8, 9^.

The use of fingerprints and the Tanimoto coefficient ^10^ (Tc), also known as the Jaccard index ^11^, represents one of the most popular methods for quantifying molecular similarity ^1– 4^. Fingerprints encode structural features of a molecule in a binary vector format and the Tc quantifies the overlap of features of two molecules as the ratio of the number of common features to the total number of features in each fingerprint. The Tc has the value range 0 to 1 and can be interpreted as the percentage of features shared by two molecules. However, whether a given percentage of overlap should be considered a significant similarity of two molecules depends on the fingerprint design and the global frequency of encoded features. Fingerprint designs might be categorized as dense or sparse. Dense fingerprints have a relatively small dimensionality of at most a few thousand features, but a significant fraction of these might be present in any given molecule. On the other hand, sparse fingerprints can have a theoretically infinite set of features (typical integer encodings allow up to 4 billion features). However, only tens or hundreds of these features might be found in a single molecule. Consequently, sparse fingerprint representations generally lead to smaller Tc values than dense fingerprints.

While it is not meaningful to compare Tc values of different fingerprint designs directly, statistical approaches can be applied to assess the significance of Tc values with respect to a reference data set. By using the distribution of Tc values obtained from comparing random compounds as a reference, Tc value significance can be determined by calculating the probability of obtaining a given Tc or higher value by chance. In statistical terms, the reference distribution corresponds to a null hypothesis and the significance measure is known as *p*-value or *p*-score. This score has the range 0 to 1 and indicates the probability that a given Tc would be obtained by chance. Thus, smaller *p*-values indicate higher significance. Here, we will use the measure 1 – ( *p*-value) to assess significance. Although it is in principle possible to obtain Tc distributions by random sampling, this process is time consuming. Instead, the ccbmlib package presented here provides methods for the generation of Tc distribution models that are based on the statistical analysis of feature frequencies and feature correlations between fingerprints for a reference data set. Some mathematical models of Tc-value distributions ^12– 14^ have been introduced in the past. The ccbmlib implementation makes use of the conditional correlated Bernoulli model (CCBM) that has been shown to accurately model Tc distributions for a variety of fingerprint designs ^13, 14^. An unconditional distribution model accounts for Tc distributions of fingerprints of randomly selected compounds. However, it is of particular interest to model distributions where one compound fingerprint is used as a reference, which forms the basis of similarity searching. *P*-values obtained from such conditional distribution models efficiently estimate how high a test compound would be ranked in a similarity search with respect to a given reference compound. Hence, conditional models can be used to predict similarity search performance ^13, 14^.

The implementation presented here is based on RDKit ^15^ and provides methods for statistically analyzing fingerprint feature distributions and building models for fingerprints implemented in RDKit. Methods are provided for calculating significance from Tc values, which enable a meaningful comparison of Tc values calculated using fingerprints of different design. The CCBM requires knowledge of the frequencies of individual features as well as their pairwise covariances. This statistical analysis needs to be carried out once for each reference data set and fingerprint design. This step can be time consuming for large data sets. The ccbmlib implementation stores resulting statistics permanently to avoid redundant calculations. For our reference implementation and evaluation, compounds from ChEMBL (release 25) ^16^ were selected as a representative sample of bioactive chemical space.

## Methods

### Fingerprint representations

RDKit provides implementations for a variety of fingerprints. Available fingerprints are reported in Table 1. The atom pair fingerprint encodes typed pairs of atoms and their bond distance and is based on the description given by Carhart and Smith ^17^, representing a sparse fingerprint. The Avalon fingerprint ^18^ is a hashed fingerprint enumerating paths and feature classes. MACCS (Molecular ACCess System) keys record the presence or absence of a dictionary of 166 substructural features ^19^. Morgan fingerprints are an RDKit implementation of extended connectivity fingerprints (ECFPs) ^20^ and enumerate atom environments up to a selected radius. We calculated Morgan fingerprints for radius 1 and 2 corresponding to ECFP with diameter 2 and 4, respectively. The topological torsion fingerprints encode sequences of four bonded atoms in a sparse fingerprint ^21^. The RDKit fingerprint is a hashed substructure/path fingerprint similar to the Daylight fingerprints ^22^. Atom pairs, Morgan fingerprints, and the topological torsion fingerprint result in sparse vector representations whose dimensions are only limited by the underlying numerical representation. Hashing is often used to yield a dense fingerprint representation of constant length. We evaluated our models using the sparse and hashed versions with a default size of 2048 bits.

**Table 1.** Fingerprints available in RDKit.

Fingerprint	Dimension	Description	*μ*( *FC*)	*σ*( *FC*)
Atom pairs	sparse	typed atom pairs	199.8	155.9
Atom pairs – hashed	2048		186.3	126.4
Avalon	512	path-based	206.3	78.9
MACCS keys	166	substructures	52.1	13.5
Morgan radius 1	sparse	atom environments	30.5	8.4
Morgan radius 1 – hashed	2048		30.1	8.2
Morgan radius 2	sparse		51.0	15.3
Morgan radius 2 – hashed	2048		50.3	14.9
Topological torsions	sparse	4-atom-paths	34.7	13.8
Topological torsions – hashed	2048		34.2	13.4
RDKit	2048	path-based	877.5	324.0

*μ*( *FC*) and *σ*( *FC*) are the average number and standard deviation of the number of features per fingerprint for ChEMBL compounds, respectively.

For the following mathematical description of the models, we will use lowercase bold letters to indicate bit vector representations and uppercase italic symbols to denote the corresponding feature set representations:

$$\begin{array}{c} a=(a_{1},a_{2},\ldots ,a_{d})\;\text{where}\;a_{i}\;\in \left\{0\text{,}1\right\},1\;\leq i\;\leq d\\ \;A=\left\{i|a_{i}=1,1\;\leq i\;\leq d\right\} \end{array}\;(1)$$

Here, *d* ∈ ℕ is the dimension of the fingerprint.

### Fingerprint similarity

Similarity of fingerprints is most often assessed on the basis of the set of features common to two fingerprints. The Tanimoto coefficient ^10, 11^ is defined as the ratio of the number of features common to two fingerprints *A* and *B* to the total number of features present in either *A* or *B*:

$$Tc\left(A,\;B\right)\;=\;\;\frac{\left|A\cap B\right|}{\left|A\cup B\right|}\;=\;\frac{I\left(A,B\right)}{U\left(A,B\right)}\;(2)$$

where *I*( *A, B*) = | *A* ∩ *B*| and *U*( *A, B*) = | *A* ∪ *B*| are the cardinalities of the intersection and union of *A* and *B*, respectively.

### Modeling similarity value distributions

The distribution of Tc values depends on the fingerprints of a reference compound data set. The resulting *p*-values must be interpreted with respect to the reference data set.

As indicated in Equation 1, fingerprints can be represented as sets of features and similarity metrics like the Tc depend on the cardinalities of the intersection and union of sets. Each of the *d* features *X _i_* of a fingerprint can be modeled as a Bernoulli variable that occurs with a certain probability *p _i_*. Given a reference data set of *N* compounds and their fingerprints *A* = { **a _*k*_**|1 ≤ *k* ≤ *N*} where **a**_*k*_ = ( *a*_*k*1_ *, a*_*k*2_ *,* … *a _kd_*) the probabilities can be estimated from the relative frequencies:

$$p_{i}\;=\;E\left(X_{i}\right)\;=\;\frac{1}{N}\Sigma_{k\;=\;1}^{N}a_{ki}\;,1\;\leq \;i\;\leq \;d\;(3)$$

The cardinality of a fingerprint itself, of the intersection, and of the union can then be modeled as a sum of non-identically distributed Bernoulli variables. In the case of independent variables, the sum follows a Poisson binomial distribution with mean

$$\mu \;=\;\Sigma_{i\;=\;1}^{d}\;p_{i}\;(4)$$

and variance

$$\sigma^{2}\;=\;\Sigma_{i\;=\;1}^{d}\;p_{i}\;\left(\text{1}\;\text{–}\;p_{i}\right)\;(5)$$

and can be approximated by a normal distribution. Because the cardinalities of the intersection and union of two sets are not independent, the Tc is then modeled as the ratio of two correlated normal distributions for which approximations exist ^23, 24^.

Fingerprint features are often correlated. Ignoring these correlations leads to a significant underestimation of the variance ( Equation 5) ^13, 14^. While the equation for the mean *μ* remains valid for correlated random variables, the formula for the variance *σ* ^2^ requires taking the pairwise covariances *c _ij_* = cov( *X _i_,X _j_*) between the different features into account. These can also be estimated from the reference set:

$$c_{ij}\;=\;E\left(\left(X_{i}\;-\;p_{i}\right)\;\left(X_{j}-p_{j}\right)\right)\;=E\left(X_{i}X_{j}\right)\;-\;p_{i}p_{j}\;=\;\frac{1}{N}\Sigma_{k\;=\;1}^{N}a_{ki}a_{kj}\;-\;p_{i}p_{j}\;(6)$$

Accordingly, the value *c _ii_* = *p _i_* (1 – *p _i_*) denotes the variance of *X _i_*.

Based on these estimates, the average cardinality of a fingerprint itself, of the intersection, and of the union of two unknown fingerprints can be determined:

$$E\left(\left|X\right|\right)\;=\Sigma_{i\;=\;1}^{d}\;p_{i}\;(7)$$

$$\mu_{I}=E\left(I\left(X,Y\right)\right)\;=\Sigma_{i\;=\;1}^{d}\;p_{i}^{2}\;(8)$$

$$\mu_{U}=E\left(U\left(X,Y\right)\right)\;=E\left(\left|X\right|\;+\;\left|Y\right|\;-\;I\left(X,Y\right)\right)\;=2\Sigma_{i\;=\;1}^{d}\;p_{i}\;-\;\Sigma_{i\;=\;1}^{d}\;p_{i}^{2}\;(9)$$

For the respective variances, one obtains:

$$\text{Var}\left(\left|X\right|\right)\;=\;\Sigma_{i\;=\;1}^{d}\;\Sigma_{j\;=\;1}^{d}\;c_{ij}\;(10)$$

$$\sigma_{I}^{2}=\text{Var}\left(I(X,Y)\right)=\Sigma_{i=1}^{d}\Sigma_{j=1}^{d}\left(c_{ij}^{2}+2c_{ij}p_{i}p_{j}\right)\;(11)$$

$$\sigma_{U}^{2}=\text{Var}\left(U(X,Y)\right)=\Sigma_{i=1}^{d}\Sigma_{j=1}^{d}2c_{ij}(1-2p_{j})+\sigma_{I}^{2}\;(12)$$

The covariance between the cardinality of union and intersection is given by:

$${\text{cov}}_{IU}=\text{Cov}\left(I(X,Y),U(X,Y)\right)=\Sigma_{i=1}^{d}\Sigma_{j=1}^{d}2c_{ij}p_{j}-\sigma_{I}^{2}\;(13)$$

Normal distributions are defined by their mean and standard deviation and can thus be calculated from the estimates of the averages and variances. However, given the fact that the underlying features are not independent, the suitability of using normal distributions as approximations cannot be guaranteed from a theoretical point of view. Nevertheless, as has been previously shown ^13, 14^, and as can be seen from our current evaluation ( *vide infra*), practical applications of the model yield good performance for a variety of different fingerprint designs. Under the assumption of normality, the following models are obtained:

$$I(X,Y)\approx N(\mu_{I},\sigma_{I}^{2})\;(14)$$

$$U(X,Y)\approx N(\mu_{U},\sigma_{U}^{2})\;(15)$$

where *N*( *μ,σ* ^2^) is the normal distribution with mean *μ* and standard deviation *σ*. The Tc distribution is then modeled as a ratio of these two correlated distributions. An analytical form of the probability distribution function exists ^23^; however, for determining *p*-values and the significance, the following approximation of the cumulative distribution function (CDF) is used ^24^:

$$F(t)\approx \Phi \left(\frac{\mu_{U}t-\mu_{I}}{\sigma_{I}\sigma_{U}a(t)}\right)\;\text{where}\;a(t)=\sqrt{\frac{t^{2}}{\sigma_{I}^{2}}-\frac{2\rho t}{\sigma_{I}\sigma_{U}}-\frac{1}{\sigma_{U}^{2}}}\;(16)$$

Here, *ρ* = cov _*IU*_/( *σ _I_σ _U_*) is the correlation between intersection and union and Φ is the CDF of the standard normal distribution:

$$\Phi (u)=\frac{1}{\sqrt{2\pi }}\int_{-\infty }^{u}\exp\left(-\frac{x^{2}}{2}\right)\text{d}x\;(17)$$

The *p*-value can then be determined as:

p=1−F(t)=Pr⁡(Tc>t)(18)

For model evaluation, we use *F*( *t*) = Pr (Tc ≤ *t*) directly as a measure of significance.

### Modeling conditional value distributions

For similarity searching, reference compounds are used and Tc values of database compounds are calculated relative to the references. As has been shown ^13^, distributions of Tc values can vary greatly depending on the reference fingerprint. In this case, the significance of Tc values should to be considered for a given reference compound. Mathematically, this corresponds to determining the conditional distributions when one fingerprint is given. As in the unconditional case, the distributions are based on sums of correlated Bernoulli variables that are modeled as normal distributions based on the conditional means and variances:

$$\mu_{I}^{A}=E\left(I\left(A,X\right)|A\right)=\Sigma_{i\in A}p_{i}\;(19)$$

$$\mu_{U}^{A}=E\left(U\left(A,X\right)|A\right)=E\left(\left|A\right|+\Sigma_{i\notin A}X_{i}\right)=\left|A\right|+\Sigma_{i\notin A}p_{i}\;(20)$$

$${\left(\sigma_{I}^{A}\right)}^{2}=\text{Var}\left(I\left(A,X\right)|A\right)=\Sigma_{i,j\in A}c_{ij}\;(21)$$

$${\left(\sigma_{U}^{A}\right)}^{2}=\text{Var}\left(U\left(A,X\right)|A\right)=\Sigma_{i,j\notin A}c_{ij}\;(22)$$

$${\mathrm{cov}}_{\text{IU}}^{A}=\mathrm{cov}\left(I\left(A,X\right),U\left(A,X\right)|A\right)=\Sigma_{i\in A}\Sigma_{j\notin A}c_{ij}\;(23)$$

The conditional model is obtained by applying these parameters in Equation 16.

A derivation of the formulas presented here for the CCBM can be found in the original publications ^13, 14^.

### Sparse fingerprints

Sparse fingerprints like ECFPs or the Morgan fingerprint might result in hundreds of thousands of different features present in large data sets. Most of these will occur with very small probabilities *p _i_* and only have a small influence on the estimated means and variances. It is computationally unproblematic to handle these individual probability estimates; however, determining pairwise covariances of all possible features becomes infeasible for more than a few thousand features. To address this issue, the complete covariance matrix is only determined for the most frequent features of a sparse fingerprint (by default, the 2048 most frequent features are selected). Covariances involving rare fingerprints are not estimated. Given that feature probabilities of combinatorial fingerprints usually show pseudo-exponential drop-offs for rare features, contributions towards covariance estimates have negligible influence on the final estimates and are ignored in the current implementation.

### Data sets

As reference data set, ChEMBL compounds were selected. SMILES representations of 1,870,461 compounds were downloaded and standardized using a previously published protocol included in the ccbmlib package ^25^. Additionally, stereochemical information was removed since most fingerprints implemented in RDKit do not account for stereochemistry, resulting in 1,691,786 unique compounds. Fingerprint statistics are reported in Table 1.

### Implementation and operation

The software has been implemented as a module for Python 3.7. It requires the installation of RDKit and has been tested with version 2019.03.4 of RDKit. Any system (Linux, Windows, MacOS) capable of running Python 3.7 and RDKit is sufficient for running our software. A 64-bit operating system with at least 8GB RAM is recommended. After obtaining the code it can be installed using Python’s setup utility. The ccbmlib package contains three modules: preprocessing, statistics, and models.

Module preprocessing consists of routines for standardizing molecules and preparing compound data sets. Standardization of molecules is a generally recommended preprocessing step, especially when compound data sets are assembled from different sources.

Module statistics contains classes for feature statistics and distribution models. Its main classes are PairwiseStats and CorrelatedNormalDistributions for the fingerprint statistics and distribution models, respectively. Distribution models are obtained from PairwiseStats objects using the get_tc_distribution method, which are used to generate unconditional and conditional models.

 The module models provides the main interface for the package. It offers wrapper functions for calculating RDKit fingerprints and contains the central method get_feature_statistics for generating or retrieving fingerprint statistics for a reference data set. Once calculated, statistics are saved and can be retrieved for later use. Exemplary applications of the module are provided in the readme file of the ccbmlib distribution.

## Results and discussion

**Figure 1.**
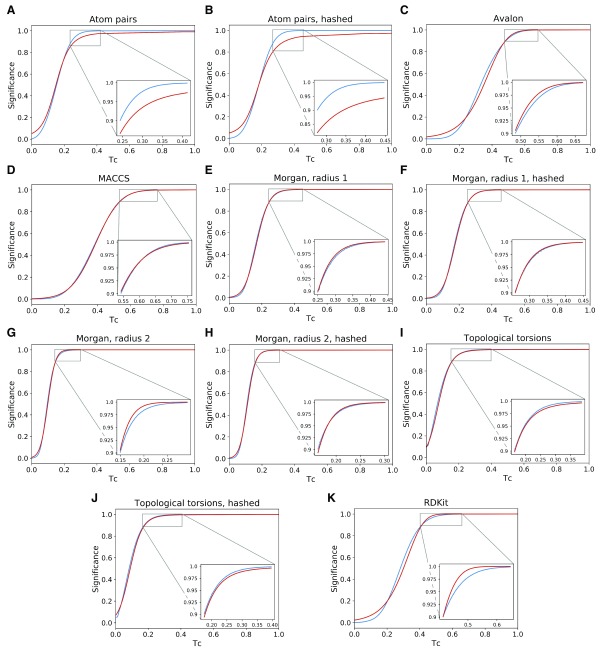
Empirical and modeled cumulative distribution functions. The empirical and modeled cumulative distribution functions for the fingerprints reported in Table 1 are shown in ( **a**) – ( **k**). Blue lines indicate empirical distributions obtained from randomly sampling 1,000,000 pairs of compounds from ChEMBL. Red lines show the corresponding modeled distributions according to Equation (16). The inserts highlight the correspondence between the curves for Tc values of high significance.

Fingerprint statistics were calculated on the basis of the 1,691,786 unique ChEMBL compounds and distribution models were derived. To evaluate the quality of the general model, 1,000,000 Tc values were calculated from pairs of random compounds drawn from the ChEMBL data set and empirical CDFs were determined. Figure 1 compares the empirical CDFs to the modeled unconditional CDFs for the fingerprints in Table 1. Overall, the modeled CDFs match the different value ranges and shapes of the empirical CDFs very well. However, to assess the usefulness of the model as a quantitative and comparative tool, the quality of the model should be assessed with a focus on Tc values indicating high significance. The insets of the figures show an enlarged section with Tc values having a significance of 0.9 or higher. The models for the atom pair fingerprints are not able to accurately model the distribution in this region. However, most other Tc distributions can be modeled very well. For the MACCS, Morgan, and topological torsion fingerprint distributions, high-quality models are obtained with small differences between the theoretical and empirical model. The hashed variants of the Morgan and topological torsion fingerprints have distributions highly similar to their sparse counterparts. This can be expected because the average feature counts reported in Table 1 are also very similar, indicating that most of the sparse features are hashed to unique values and only few collisions occur between hashed values. The path-based Avalon and RDKit fingerprints still have usable, although less accurate models. These observations are consistent with previous observations ^13^. CCBM models pharmacophore-based fingerprints only to a limited extent. This might be due to the specific nature of correlations between pharmacophore features.

A quantitative summary of the observations is given in Table 2. It reports the Kolmogorov-Smirnov statistic (KS) ^26^, which is defined as the maximum difference between empirical ( *F*_emp_) and modeled ( *F _model_*) distributions:

$$\text{KS}\left(F_{\text{emp}},F_{\text{model}}\right)=\max_{x}\left|F_{\text{emp}}\left(x\right)-F_{\text{model}}\left(x\right)\right|\;(24)$$

**Table 2.** Kolmogorov-Smirnov statistics.

Fingerprint	KS	KS _90_
Atom pairs	5.47%	4.22%
Atom pairs – hashed	8.80%	8.80%
Avalon	6.91%	1.04%
MACCS	2.09%	0.43%
Morgan radius 1	3.64%	0.54%
Morgan radius 1 – hashed	3.37%	0.30%
Morgan radius 2	4.16%	1.26%
Morgan radius 2 – hashed	3.80%	0.83%
Topological torsions	9.31%	0.47%
Topological torsions – hashed	6.78%	0.75%
RDKit	8.03%	1.70%

KS reports the Kolmogorov-Smirnov statistic comparing the experimental to the modeled distributions. KS _90_ reports the Kolmogorov-Smirnov statistic limited to Tc values with an empirical significance of at least 90%.

In addition, the maximum difference for the significance range beyond 90% is reported (KS _90_):

$${\text{KS}}_{90}(F_{\text{emp}},F_{\text{model}})=\max_{x,F_{\text{emp}}(x)\geq 0.9}|F_{\text{emp}}(x)-F_{\text{model}}(x)|\;(25)$$

The maximum difference for most models is observed for common Tc values, i.e., where the slope of the CDF is steepest. However, as can be seen from the KS _90_ values, the high significance range can be accurately assessed within 1% for MACCS, most Morgan, the torsion, and the Avalon fingerprints. The RDKit fingerprint still performs reasonably well with a KS _90_ of 1.70, whereas values of 4.22 and 8.80 for the atom pair fingerprint and its hashed variant indicate poor performance of the model in this region.

**Figure 2.**
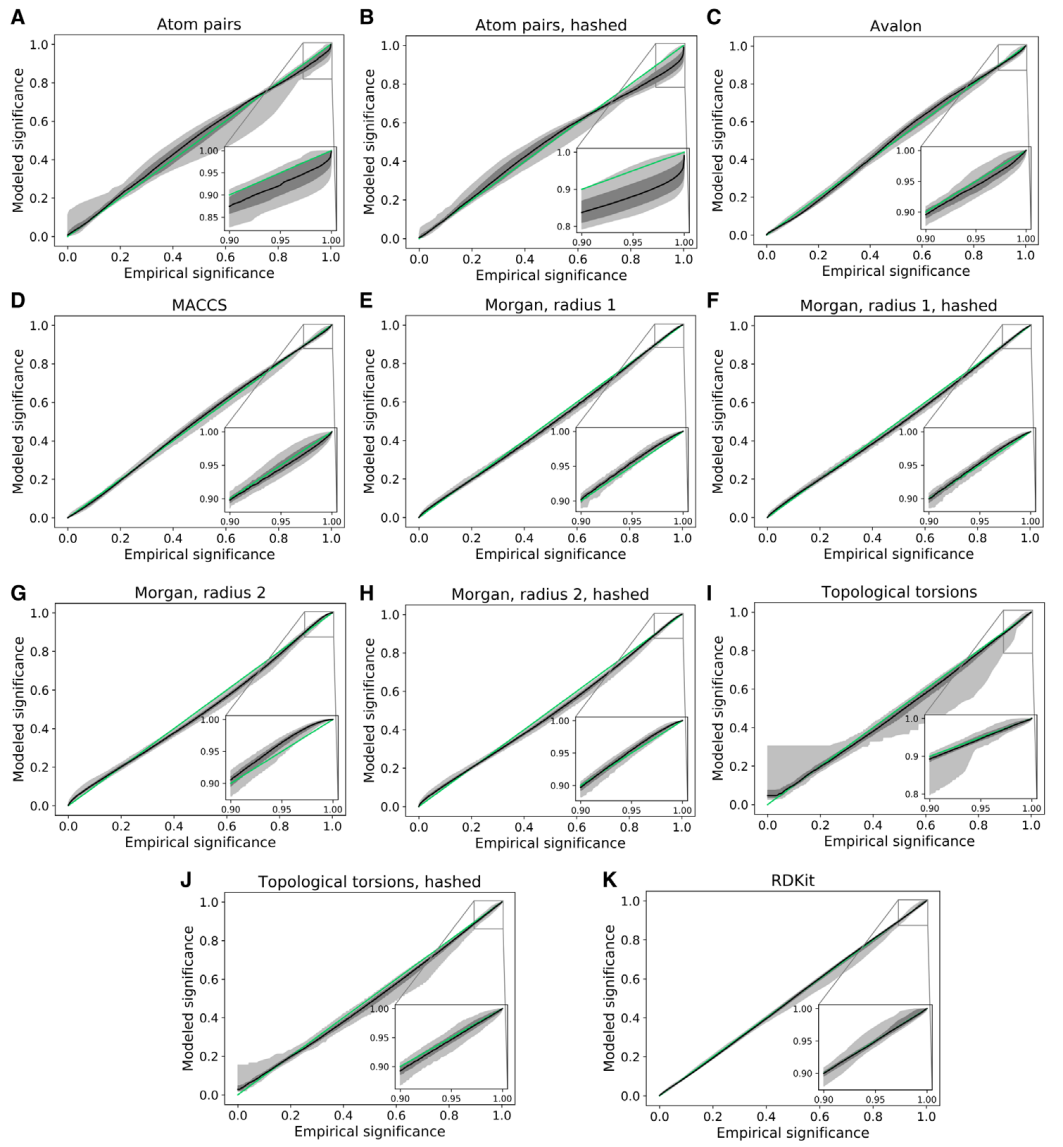
Empirical versus modeled significance values. For the fingerprints in Table 1, each of the graphs ( **a**) – ( **k**) shows the variation of correspondences between empirical and modeled significance values of 100 conditional distributions obtained by selecting random reference compounds. Empirical distributions for each reference compound were determined from comparisons of 100,000 randomly chosen compounds. The black line indicates the median correspondence between empirical and modeled distribution. The dark gray area shows the interquartile range and the light gray area the range from the 5 ^th^ to the 95 ^th^ percentile. The green line is the diagonal corresponding to a perfectly matching model. The inserts highlight correspondences for significance values larger than 0.9.

In addition to the unconditional model, conditional distributions were investigated when a reference fingerprint was given. As each reference fingerprint will yield a different model, 100 compounds were randomly chosen as a reference and conditional models were derived and compared to empirical Tc distributions by comparing the reference compound to 100,000 randomly chosen compounds. The ranges of correspondences between empirical and modeled significance values are shown in Figure 2. The MACCS and Morgan fingerprints again showed the best conditional models, all of which were close to the ideal diagonal. For most reference compounds, the topological torsion fingerprint also yielded very good models; however, few outliers with large deviations were observed. This might be expected when reference fingerprints only contain very few features and approximations by normal distributions fail to yield accurate models.

**Figure 3.**
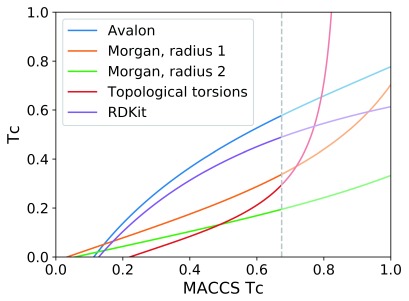
Corresponding Tc values for fingerprints of different design. The graphs show the relation of MACCS Tc values to Tc values of other fingerprint designs with corresponding significance scores. The dashed line corresponds to a significance of 0.99.

The modeled unconditional CDFs can be used to relate Tc values of different fingerprints to each other by determining the significance score for one type of fingerprint and using the inverse CDF to identify the corresponding Tc value of another fingerprint design. A caveat here is that for very high significance scores the CDF essentially becomes a flat line and thus the inverse would not be well defined. Figure 3 shows the correspondence between MACCS Tc values and Tc values of other fingerprint designs. The graphs emphasize how differently Tc values of different fingerprint designs have to be interpreted. For instance, a MACCS Tc 0. 60 corresponds to a Morgan, radius 2 Tc of 0.17 and an RDKit Tc of 0.45, each indicating a significance score of around 0.96. The vertical dashed line corresponds to a significance of 0.99 beyond which he curves are expected to be less reliable and have been grayed out accordingly.

The Python code used for data generation, data analysis, and generation of the figures is available in form of a Jupyter notebook in the GitHub repository ^27^.

## Conclusions

The tools provided make it possible to evaluate the significance of Tc values for a variety of fingerprints from RDKit. Users can generate distribution models for different fingerprints with respect to reference data sets. Accurate models are obtained for most RDKIT fingerprints including the popular MACCS and Morgan fingerprints. Based on these models, it can be assessed to what extent molecular similarity is accounted for by fingerprints of different design and to what extent similarity between compounds sharing the same activity is reflected by similarity scores calculated on the basis of different fingerprint representations. Furthermore, the conditional models can be used to predict the suitability of fingerprints for similarity searching and ligand-based virtual screening.

## Data availability

### Source data

The data sets used in this paper are freely available from ChEMBL: https://www.ebi.ac.uk/chembl/

Smiles structure representations were retrieved on 15 Jan 2020 from: ftp://ftp.ebi.ac.uk/pub/databases/chembl/ChEMBLdb/latest/chembl_25_chemreps.txt.gz

## Software availability

### RDKit

Our package depends on RDKit, which is freely available from https://www.rdkit.org

### ccbmlib

Source code is available from: https://github.com/vogt-m/ccbmlib

Archived source code at time of publication: https://doi.org/10.5281/zenodo.3691943 ^27^

License: MIT
