# Supplementary material for: ccbmlib – a Python package for modeling Tanimoto similarity value distributions
Source: F1000Res. 2020 Mar 5;9:Chem Inf Sci-100. Originally published 2020 Feb 10. [Version 2] doi: 10.12688/f1000research.22292.2 (PMC7050271; doi:10.12688/f1000research.22292.2)
Supplement: Peer review history

# Peer review: Reviewer response for version 1

**DOI:** 10.5256/f1000research.24591.r59805

**Published:** 2020-02-28

**Authors:** David A. Cosgrove

**Affiliations:** 1 CozChemix Limited, Macclesfield, UK

The authors report a method for analysing the occurrence of features in a set of fingerprints that have been generated from a reference collection of chemical structures. They use this analysis to generate models for assessing the statistical significance of the tanimoto coefficients for pairs of fingerprints in the set. Using the model, they can produce a plot of significance vs tanimoto coefficient (a CDF). In the paper, the accuracy of the model is assessed by comparing the curve so produced with those created by calculating the tanimoto coefficients for pairs of fingerprints from a large random sample of the set. The correspondence between the modelled and empirical distribution functions is high.

 The paper is clearly laid out and relatively easy to read, if one takes the maths at face value. It is likely that it would be possible to reproduce their analysis from the information given. However, that is not strictly necessary from a practical standpoint as the authors have made the software they have developed for the analysis available as a Python module for anyone to download and use. They are to be commended for this action, which is still rare in the field of cheminformatics. It is likely to increase the impact of the paper considerably.

 When I read a paper of this nature, a key question I pose myself is “how, if at all, will this help me with my work?” Here I fear the authors have been less successful. For example, there is an implementation in the RDKit toolkit of the Taylor-Buttina clustering method. This is a popular way of clustering fingerprints, and hence molecules, that is widely used for things like analysis of high-throughput screening results, organising the results from a virtual screen etc. A key input parameter to the algorithm is a threshold tanimoto coefficient – all fingerprints within a cluster are guaranteed to be within this similarity of the first fingerprint placed in the cluster. The success of this method for clustering depends very strongly on the value chosen for this threshold. Too high, and one obtains an unhelpfully large number of small clusters; too low, and the clusters will be large and contain molecules without apparent similarity. It would be very useful if there were a way of taking a successful threshold for one fingerprint type and using it to decide upon a similarly successful threshold for a different type. I feel as though this paper contains a way of doing this, but it is unclear to me quite how it would be achieved with the results presented. If the authors could add to the paper an example of how one would take a CDF for one fingerprint type and use it to translate a useful tanimoto coefficient threshold for it into an equally useful threshold for a different fingerprint type, that would, in my opinion, make the paper much more valuable.

I confirm that I have read this submission and believe that I have an appropriate level of expertise to confirm that it is of an acceptable scientific standard.

# Response

**Published:** 2020-02-29

**Authors:** Jürgen Bajorath

**Affiliations:** LIMES Program Chem. Biol. & Med. Chem, University of Bonn, Germany

Thank you for your comments and your suggestion. Indeed, a potential application of the methodology is establishing correspondences between Tc values of different fingerprints according to their statistical significance. Therefore, a paragraph has been added to the manuscript explaining how modeled distributions can be used to identify corresponding Tanimoto coefficients (Tc values) for fingerprints of different design. In addition, a figure has been added displaying the relationship between MACCS Tc values and Tc values of other fingerprints. The software and Jupyter notebook have been updated accordingly.

# Peer review: Reviewer response for version 1

**DOI:** 10.5256/f1000research.24591.r59806

**Published:** 2020-02-28

**Authors:** Brian Goldman

**Affiliations:** 1 Modeling & Informatics, Vertex Pharmaceuticals, Boston, MA, USA

The article ‘ccbmlib: a Python package for modeling Tanimoto similarity value distributions’, by Vogt and Bajorath is clearly written and concretely describes a method for determining the significance of tanimoto similarity scores. The statistical technique detailed in the paper outlines a mathematical method for converting tanimoto similarity scores from various binary molecular fingerprints into significance (p) values. Consequently, the method provides a way of normalizing similarity scores so that comparisons between results of searches utilizing different fingerprinting methods can be conducted easily. The paper also outlines a ‘conditional method’ that provides a technique for estimating the distributions of similarity scores for a given reference compound. This allows one to estimate how well a test compound would rank in a large-scale similarity search.

 The explanations and mathematical equations in the paper are easy to follow. The graphs in the results section clearly support the findings of the study. I would recommend this paper to be indexed in its current form.

I confirm that I have read this submission and believe that I have an appropriate level of expertise to confirm that it is of an acceptable scientific standard.

# Response

**Published:** 2020-02-29

**Authors:** Jürgen Bajorath

**Affiliations:** LIMES Program Chem. Biol. & Med. Chem, University of Bonn, Germany

Thank you for your instructive comments on the manuscript.
